# Orchestrating synaptic strength by neuroligin-confined nanoscale organization

**DOI:** 10.1016/j.mocell.2026.100319

**Published:** 2026-01-16

**Authors:** Ying Han, Xiaoyan Dai, Bo Zhang

**Affiliations:** 1 Institute of Neurological and Psychiatric Disorders, Shenzhen Bay Laboratory, Shenzhen 518132, China; 2 School of Chemical Biology and Biotechnology, Peking University Shenzhen Graduate School, Shenzhen 518055, China; 3 Shenzhen Medical Academy of Research and Translation, Shenzhen 518132, China

**Keywords:** Cell adhesion molecules, Nanocluster, Neuroligin, Synapse

## Abstract

The synaptic nanocluster refers to the nanoscale accumulation of synaptic proteins that ensures the fidelity of synaptic transmission. These nanoclusters align pre- and post-synaptic machineries across the synaptic cleft, enabling neurotransmitter release to occur precisely opposite postsynaptic receptors. In vertebrates, neuroligins are key postsynaptic cell adhesion molecules that help maintain synaptic function. Recent superresolution microscopy studies have shown that neuroligins themselves form nanoclusters and regulate the nanoscale distribution of synaptic proteins, suggesting a role in orchestrating synaptic strength through nanocluster organization. In this review, we summarize recent advances in understanding how neuroligin subtype specificity influences the organization of postsynaptic receptors and how neuroligin-ligand interactions modulate nanoscale architecture. We also discuss how disruption in those nanoclusters can disturb synaptic strength.

## Graphical Abstract

**Figure:**
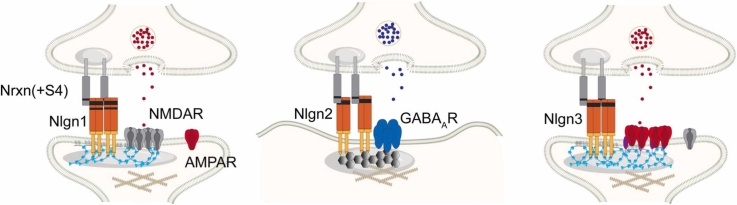

## INTRODUCTION

Chemical Synapses are the specialized connections between neurons. These synapses are highly compartmentalized, submicron-sized structures. Recent advances in imaging and computational analysis have been essential for visualizing and quantifying the synapse’s nanoarchitecture. Electron microscopy has revealed electron-dense regions in the presynaptic and postsynaptic sites, known as the active zone (AZ) and postsynaptic density (PSD), respectively (Acuna et al., 2016, Dosemeci et al., 2016). With the development of superresolution techniques, such as stochastic optical reconstruction microscopy (STORM) and 10× expansion microscopy, researchers have identified intricate yet highly ordered nanoscale organizations of synaptic proteins, including Munc13-1, PSD-95, and alpha-amino-3-hydroxyl-5-methyl-4-isoxazolepropionic acid receptors (AMPARs), which form clusters in nanoscale (Chen et al., 2015, Fukata et al., 2013, MacGillavry et al., 2013, Nair et al., 2013, Nozawa et al., 2022, Sigal et al., 2018, Tang et al., 2016). N-methyl-D-aspartate receptors (NMDARs) also form nanoclusters, and a subset of release sites is located near NMDAR nanoclusters (Anderson et al., 2025). Nanoclusters of AZ and PSD scaffolds have been observed under near-native conditions using focused-ion beam milling and cryo-electron tomography (Held et al., 2024). The precise transsynaptic alignment of these nanoclusters enhances the efficiency and fidelity of synaptic transmission (Biederer et al., 2017, Xu et al., 2025). However, under resting conditions, synaptic vesicles and PSD-95 nanoclusters are not well aligned (Held et al., 2024); alignment likely improves during action potential-triggered activation. Moreover, different synapse types may employ slightly varied nanocluster arrangements to fine-tune their specific function, as seen in 2 types of excitatory hippocampal synapses with distinct Munc13-1 and PSD-95 nanocluster patterns (Dharmasri et al., 2024). Inhibitory synaptic machineries can also be recognized as distinct nanoclusters on the plasma membrane of somata, dendrites, and axon initial segments in the hippocampal CA1 pyramidal cells (Kasugai et al., 2010).

The nanoscale organization of synapses may be shaped by synaptic cell adhesion molecules (CAMs), which play key roles in regulating synaptic connectivity between neurons (Biederer et al., 2017, Xu et al., 2025). Those CAMs, including neurexins (Nrxns), neuroligins (Nlgns), Rab3-interacting molecule (RIM), and leucine-rich-repeat transmembrane proteins (LRRTMs), are not evenly distributed but instead form discrete transcellular nanocolumns (Chamma et al., 2016, Han et al., 2022, Igarashi et al., 2018, Lloyd et al., 2023, Nozawa et al., 2022, Ramsey et al., 2021, Tang et al., 2016). The broader role of synaptic strength in relation to nanoscale alignment and dynamic molecular organization has been extensively reviewed elsewhere (Biederer et al., 2017, Chamma and Thoumine, 2018, Choquet and Triller, 2013, Dharmasri et al., 2024, Xu et al., 2025). In this review, we will specifically focus on how Nlgns organize the nanoscale arrangement of postsynaptic receptors, how modulatory factors influence Nlgns and their associated complexes, and how phase separation contributes to the formation of nanoclusters.

Nlgns are postsynaptic CAMs that bind to presynaptic Nrxns (Ichtchenko et al., 1996, Südhof, 2017). They are type I transmembrane proteins encoded by 5 genes in humans (*NLGN1, NLGN2, NLGN3, NLGN4X, and NLGN4Y*) and 4 in rodents (*Nlgn1-4*). Structurally, all Nlgns share a similar domain organization: an N-terminal esterase homology domain, a conserved transmembrane domain, and a short cytoplasmic tail containing the PDZ (Postsynaptic density 95; Discs large; Zonula occludens-1) binding domain (Bourne and Marchot, 2014, Ichtchenko et al., 1996). Despite their structural similarity, Nlgns exhibit distinct synaptic localization patterns that underlie their diverse functional roles: Nlgn1 locates mainly found at glutamatergic excitatory synapse; Nlgn2 almost exclusively localizes to GABAergic inhibitory synapses; Nlgn3 is present at both types; and Nlgn4 shows a preference for glycinergic synapses (Ali et al., 2020, Altas et al., 2024, Bourne and Marchot, 2014, Budreck and Scheiffele, 2007, Ducrot et al., 2025, Hoon et al., 2011, Song et al., 1999, Varoqueaux et al., 2004, Wang et al., 2025, Zhang et al., 2018). In the following section, we will discuss the properties of Nlgn subtypes, their binding partners, and the mechanisms by which they regulate nanoscale organization.

## SUBTYPE-SPECIFIC FUNCTION OF Nlgns ON NANOARCHITECTURE OF SYNAPTIC RECEPTORS

### Neuroligin 1

**Fig. 1:**
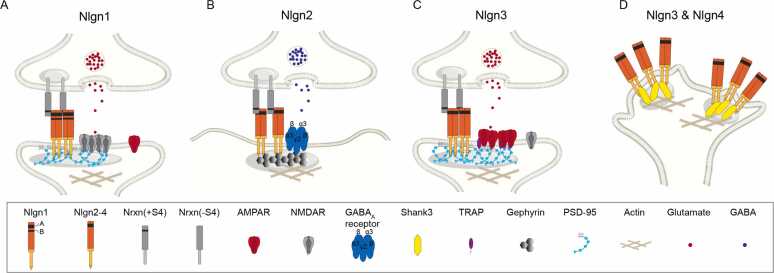
Synaptic distribution of Nlgn subtypes and associated nanoclustered molecules. All neuroligins share a conserved overall architecture: an extracellular esterase homology domain, a transmembrane domain, and a short cytoplasmic tail. The N-terminal domain forms a dimer with another neuroligin and binds presynaptic neurexins. At the same time, the cytoplasmic part contains a PDZ-binding motif that interacts with PSD-95 or gephyrin to organize the synaptic component. All neuroligins include splice site A, but only Nlgn1 also contains splice site B. (A) Nlgn1 is associated with NMDAR, but not AMPAR, in vivo. (B) Nlgn2 is restricted to GABAergic synapses, where it clusters α3 and *γ*2 subunits of GABA_A_ receptors. (C) Nlgn3 specifically clusters AMPAR, but not NMDARs, at the calyx of Held synapse. (D) Both Nlgn3 and Nlgn4 form nanoclusters at the edge of growth cones.

Most genetic evidence indicates that Nlgn1 is essential for NMDAR-mediated synaptic transmission. Knockout of Nlgn1 selectively reduces NMDAR-mediated synaptic responses in multiple brain regions, including the hippocampus, cortex, and cerebellum (Jiang et al., 2017, Zhang and Südhof, 2016). A splicing isoform of Nlgn1 containing the B insert regulates postsynaptic NMDAR level at thalamocortical synapses through interaction with Nrxn1α and astrocyte-derived Hevin (Singh et al., 2016). Consistent with its role in excitatory synapses, STORM imaging of overexpressed Nlgn1 in cultured hippocampal neurons revealed a strong spatial correlation between Nlgn1 nanoclusters and excitatory synapses (Haas et al., 2018, Hosokawa et al., 2021). The point mutation that substituted a cysteine for arginine 451 in Nlgn3 (Arg451Cys-Nlgn3) was found in siblings with autism spectrum disorder (ASD). The Arg473Cys-Nlgn1 mutation, the rat homolog of the Arg451Cys-Nlgn3 mutation, was transported to the neuronal surface as wild-type Nlgn3, even though with some cytoplasmic retention, and the overexpression of Arg473Cys-Nlgn1 led to a dramatic increase in NMDAR-mediated currents, along with enhanced clustering of NMDARs (Fig. 1A) (Khosravani et al., 2005). The different effects of wild-type and mutant Nlgn1 on NMDAR clustering suggest that the loss of Nrxn binding in the Arg473Cys-Nlgn1 variant alters NMDAR distribution. Live-cell and superresolution imaging further showed that Nlgn1 remains at synapses much longer than expected based on extracellular Nrxn interactions alone, implying that intracellular scaffolding proteins help anchor Nlgn1 at postsynaptic sites (Lagardère et al., 2022). These findings suggest that Nlgn1 is stabilized at the synapse through multivalent interactions with intracellular scaffolds and signaling pathways (Szíber et al., 2024).

The endogenous localization and nanoclustering of Nlgn1 have been revealed using 2 independent knock-in mouse lines. In the first, an HA epitope was inserted into the Nlgn1 gene, and HA-Nlgn1 was found to form nanoclusters primarily in excitatory synapses, where it likely acts as a structural unit alongside PSD-95 (Nozawa et al., 2022, Nozawa et al., 2018). In the second, Nlgn1 was N-terminally tagged with a biotin acceptor peptide and also formed nanoclusters at excitatory synapses (Ducrot et al., 2025). Surprisingly, endogenous Nlgn1 was not restricted to excitatory synapses: the HA-tagged line detected HA signals in the cerebellar pinceau, a specialized structure formed by axons of cerebellar molecular layer interneurons and astrocytes, while the biotin acceptor peptide-tagged line showed Nlgn1 nanoclusters colocalizing with gephyrin in inhibitory synapses (Ducrot et al., 2025). The function of Nlgn1 at those non-excitatory synapses remains unclear, but it may contribute to neuronal processes beyond synaptic transmission.

### Neuroligin 2

Nlgn2 is localized explicitly to GABAergic postsynapses, where it clusters GABA_A_ receptor subunits α3 and *γ*2, reflecting its functional specificity for inhibitory synapses (Fig. 1B) (Hoon et al., 2009, Kasugai et al., 2010). Notably, the absence of gephyrin and GABA_A_ α1 does not prevent the initial formation of postsynaptic specialization or Nlgn2 clustering (O’Sullivan et al., 2009, Patrizi et al., 2008). In contrast, loss of Nlgn2 disrupts GABAA receptor clustering, impairs the formation of postsynaptic structures, and impairs inhibitory transmission (Hoon et al., 2009, Liang et al., 2015, Poulopoulos et al., 2009). Together, these findings highlight Nlgn2 as a central organizer that coordinates the nanoscale assembly of synaptic proteins at inhibitory synapses.

High-resolution imaging using 3D superresolution structured illumination microscopy (3D-SIM) and STORM further shows that Nlgn2 forms nanoclusters at inhibitory synapses, which are closely associated with gephyrin (Gookin et al., 2022). Rapid removal of Nlgn2’s extracellular domain leads to nanoscale dispersion and mislocalization of RIM1/2 and GABA_A_ receptors, impairing inhibitory transmission (Xu et al., 2024). These results suggest that Nlgn2 sustains inhibitory synaptic transmission by stabilizing nanoclusters of key synaptic proteins at GABAergic synapses.

### Neuroligin 3/4

The mutation or deletion of Nlgn3 has been associated with ASD (Altas et al., 2024, Budreck and Scheiffele, 2007, Uchigashima et al., 2021). Nlgn3 specifically regulates AMPAR-mediated, rather than NMDAR-mediated, synaptic transmission at the calyx of Held (Fig. 1C) (Han et al., 2022). In Nlgn3 knockout mice, AMPAR-mediated transmission shows reduced amplitude and slower kinetics. Superresolution microscopy and computational modeling further revealed that these phenotypes result from increased nanocluster volume but decreased density of GluAs and PSD-95 clusters (Han et al., 2022). Using a specific antibody, Nlgn3 is detected at both glutamatergic and GABAergic synapses, with its distribution influenced by distinct phosphorylation states (Altas et al., 2024). Through the CRISPR/Cas9 technique, an HA-tag was inserted at the N-terminus of Nlgn3, and endogenous Nlgn3 is detected at both excitatory and inhibitory synapses (Qin et al., 2025). Similarly, genetic deletion of Nlgn4 reduces the number of glycinergic receptors and slows glycinergic miniature inhibitory postsynaptic currents (Hoon et al., 2011, Zhang et al., 2018). Together, these findings suggest that Nlgn3 and Nlgn4 are functionally linked to the nanocluster organization of glutamate and glycine receptors, respectively. Interestingly, Nlgn3 is also robustly expressed in astrocytes, where it forms clusters that may influence transcription, at least in the cerebellum (Qin et al., 2025). Additionally, both Nlgn3 and Nlgn4X form nanoclusters at the edge of growth cones in human neurons (Fig. 1D) (Gatford et al., 2022), although their roles in vivo remain unclear.

## MODULATORS OF SYNAPTIC Nlgn-Nrxn NANOSTRUCTURE

### Nrxn Nanoclusters

Nrxns, the binding partners of Nlgns, exist as alpha- and beta-isoforms and undergo alternative splicing at 6 sites, generating thousands of possible isoforms (Südhof, 2017). Nrxn1 forms discrete nanoclusters at excitatory synapses, and both the amount of Nrxn1 per synapse and the proportion of synapses containing Nrxn1 nanoclusters increased during development, as observed in hippocampal cultures and HA-Nrxn1 knockin mouse tissues (Trotter et al., 2019). Ectodomain cleavage of Nrxn1 by ADAM10 dynamically regulates the size of Nrxn1 nanoclusters and enhances neuronal activity, thereby shifting their localization from the center to the periphery of the AZ (Trotter et al., 2019). This dynamic redistribution suggests that neuronal activity actively reorganizes the positioning of Nrxn1 nanoclusters. Nrxn1 and Nrxn3 differentially regulate NMDAR- and AMPAR-mediated currents at the same hippocampal synapses, respectively (Aoto et al., 2013, Dai et al., 2019). A potential underlying mechanism is that endogenous Nrxn1 and Nrxn3 form distinct, non-overlapping nanoclusters that selectively interact with their respective postsynaptic ligands (Lloyd et al., 2023).

### Ligands of Nrxn on Nlgn Nanoclusters

**Fig. 2:**
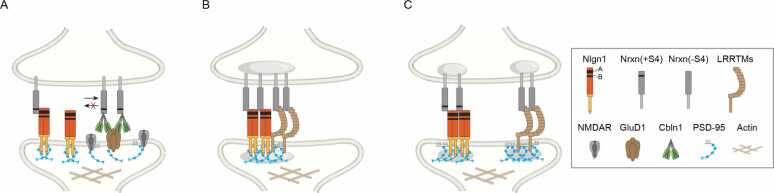
Competition and cooperation among Nlgns and synaptic modulators. (A) Cbln1 competes with Nlgn 1 for binding to presynaptic Nrxn(+S4) at parallel fiber-interneuron synapses. (B) In hippocampal CA3 synapses, Nlgn1 and LRRTM1 form overlapping nanoclusters, both associating with presynaptic Nrxn(-S4). (C) In cortical synapses, Nlgn1 and LRRTM1 form distinct nanoclusters, interacting with presynaptic Nrxn(+S4) and Nrxn(-S4), respectively.

The interactions between Nlgns and Nrxns are shaped by alternative splicing of both proteins (Araç et al., 2007, Südhof, 2017). For example, the extracellular scaffolding protein cerebellin 1 (Cbln1) binds explicitly to presynaptic Nrxn containing splice site 4 insert (Nrxn(+S4)), but not Nrxn lacking this insert (Nrxn(-S4)), along with the postsynaptic receptor GluD1 at synapses between cerebellar parallel fibers and molecular layer interneurons (Fig. 2A) (Nozawa et al., 2022). In the cerebellar cortex, Cbln1 modulates the size and number of Nlgn1 nanoclusters by competing for binding to presynaptic Nrxn(+S4) (Matsuda and Yuzaki, 2011, Nozawa et al., 2022). Similarly, adding exogenous Cbln1 to hippocampal neurons suppresses the recruitment of NMDARs and PSD-95 in dendrites, indicating that Cbln1 inhibits the clustering of functional NMDARs by competing with Nlgn1 for Nrxn(+S4) binding (Nozawa et al., 2022). This competitive interaction among ligands highlights a dynamic, activity-dependent regulation of Nlgns and their associated postsynaptic receptors.

LRRTMs are a group of type 1 transmembrane proteins that interact with presynaptic Nrxns and postsynaptic PSD-95 (Ko, 2012, Südhof, 2017). Specifically, LRRTM1/2 bind to Nrxn(-S4) but not to Nrxn(+S4) (Ko et al., 2009, Siddiqui et al., 2010). Using superresolution and live-cell imaging, Nrxn1β, Nlgn1, and LRRTM2 have been observed to accumulate at synapses in cultured hippocampal neurons. At the same time, Nlgn1 forms dynamic nanoclusters, and LRRTM2 forms relatively stable, compact nanoclusters in response to synaptic stimulation (Chamma et al., 2016). By leveraging expansion microscopy and HA-Nlgn1 knock-in mice, Nlgn1 and LRRTM1 nanoclusters were found to overlap at synapses in the hippocampal CA3 region, where Nrxn(-S4) is enriched (Fig. 2B) (Nozawa et al., 2022). In contrast, in the cerebral cortex, Nlgn1 and LRRTM1 nanoclusters do not overlap, likely due to the co-existence of presynaptic Nrxn with and without the site 4 insert (Fig. 2C) (Nozawa et al., 2022). In the cortical layer 5a synapses, NMDAR nanoclusters near Nlgn1 tend to be larger, whereas AMPAR nanoclusters are positioned closer to LRRTM1 (Nozawa et al., 2022). At the calyx of Held synapse, downregulation of Cbln1 permits AMPAR nanocluster formation to be regulated by Nlgn3 (Chanda et al., 2017, Han et al., 2022). Together, these findings suggest that presynaptic Nrxn isoforms, developmental stages, or related processes determine the composition of postsynaptic Nlgn nanoclusters.

## Nlgn NANOCLUSTERS TUNE SYNAPTIC STRENGTH

The maintenance of nanoclusters is crucial for synaptic transmission. Nlgn1 knockout selectively reduces NMDAR-mediated currents (Espinosa et al., 2015, Hoy et al., 2013, Jiang et al., 2017, Jung et al., 2010, Wu et al., 2019, Zhang and Südhof, 2016), likely through binding to NMDARs directly (Budreck et al., 2013) or interacting with PSD-95 indirectly. In contrast, overexpression of Nlgn1 in cultured neurons significantly enhances AMPAR clustering (Haas et al., 2018), yet Nlgn1 deletion does not impair AMPAR-mediated synaptic transmission at the calyx of Held in vivo (Han et al., 2022). Nlgn3 localizes at both excitatory and inhibitory postsynaptic sites (Budreck and Scheiffele, 2007), and its conditional deletion reduces excitatory synaptic transmission at both the calyx of Held and cerebellar climbing fiber synapses (Zhang et al., 2017, Zhang et al., 2015). Computational simulations suggest that the amplitude of miniature excitatory postsynaptic currents (mEPSCs) depends strongly on the spatial relationship between presynaptic release sites and AMPAR nanoclusters: release events occurring outside clustered AMPAR regions are predicted to generate very small, and likely undetectable, mEPSCs, which are referred to as “non-cluster” events (Han et al., 2022, MacGillavry et al., 2013, Nair et al., 2013). As a result, mEPSC measurements provide a direct readout of how synaptic adhesion molecules influence the number and distribution of AMPAR nanoclusters. At the calyx of Held, parvalbumin (Pv)-Cre-mediated conditional deletion of Nlgn3 in postnatal day 12-13 mice reduced mEPSC amplitudes and slowed kinetics (Zhang et al., 2017), suggesting altered AMPAR nanocluster distribution. Simulations further show that clustering of existing AMPARs, regardless of initial geometry, can substantially potentiate AMPAR currents (Freche et al., 2011, Haas et al., 2018). More recent work indicates that Nlgn3 directly regulates AMPAR nanoclusters, likely by modulating PSD-95 clustering (Han et al., 2022). Thus, these findings suggest that Nlgn3 helps shape AMPAR nanoclusters during development, a process that an ASD-associated Nlgn3 mutation, R451C, may disrupt (Chanda et al., 2016).

## NANOCLUSTER DIVERSITY TUNED BY Nlgns

The size, content, and position of nanoclusters are not static but are dynamically regulated by development and neuronal activity, playing a key role in synaptic plasticity. Superresolution quantitative analysis of synaptic ultrastructure revealed that the average diameters of nanoclusters in the AZ, PSD, and AMPAR are approximately 80 nm, with each hippocampal synapse in culture typically containing 3-5 PSD and AZ nanoclusters (Held et al., 2024, MacGillavry et al., 2013, Tang et al., 2016). On average, each synapse houses about 8 docked vesicles, and a positive correlation exists between the number of AZ nanoclusters, but not PSD nanoclusters, and the number of membrane-proximal vesicles (Held et al., 2024). The projected distance between AZ and PSD nanoclusters is around 36 nm, which is shorter than expected for a random distribution. In contrast, the spacing between AZ nanoclusters and membrane-proximal vesicles does not differ from that expected for random placement (Held et al., 2024), suggesting activity-dependent regulation of vesicle release.

In calyx synapses, deletion of Nlgn3 does not alter the number of PSD-95 nanoclusters per synapse. Still, it leads to larger (increased by 40%-45%) and less dense (decreased by 45%-50%) nanoclusters of PSD-95 and GluA1, without affecting presynaptic RIM1 (Han et al., 2022). Those data indicate that Nlgn3 helps confine AMPARs within nanoclusters, and its loss allows lateral diffusion of postsynaptic proteins away from the release site, resulting in reduced synaptic response amplitude and slower kinetics (Han et al., 2022). Similarly, *Drosophila* Nlgn1 (dNlgn1) forms discrete clusters adjacent to postsynaptic GluAs and supports postsynaptic differentiation and growth at neuromuscular junctions (Banovic et al., 2010). In a dNlgn1 mutant that interrupts Nrx1 binding, the T-bar-shaped AZs become star-shaped, and the diameter of the AZ scaffold protein increases from 0.233 to 0.305 µm (Owald et al., 2012), suggesting that dNlgn1 is indispensable for organizing pre- and post-synaptic nanoclusters.

## NANOCLUSTERS AND PHASE SEPARATION

The stability of synaptic protein nanoclusters largely depends on interactions among the constituent proteins. These nanoclusters exhibit a higher local concentration of molecules than the surrounding environment, even in the absence of a distinct boundary (Wu et al., 2025), suggesting a more fluid and dynamic organizational mechanism. Purified scaffolding proteins, including presynaptic RIM and Munc13, as well as postsynaptic PSD-95, Shank3, and Homer3, interact with each other and form condensates through liquid-liquid phase separation both in solution and on membrane surfaces (Chen et al., 2022, Wu et al., 2023). More recently, PSD-95 and Shank3 form glass-like condensates via phase separation (Jia et al., 2025). Shank3 oligomerization leads to rigid, plastic PSD condensates, whereas Shank3 mutations soften PSD condensates and impair synaptic plasticity (Jia et al., 2025). Such condensates may not only stabilize nano-organized receptor assemblies but also compartmentalize enzymes needed for downstream signal amplification (Wu et al., 2025). It is reasonable to propose that phase separation-driven condensates help maintain the organization of synaptic proteins, with Nlgns potentially anchored to PSDs within those membraneless assemblies. However, it remains unclear whether and how those condensates contribute to CAM-regulated nanocluster formation.

Phase separation requires high protein concentration and forms large condensates in vitro. In contrast, the superresolution studies reveal hundreds of copies of proteins within nanoclusters (Han et al., 2022). The key is the local density within the synaptic nanoclusters rather than the bulk concentration in phase separation. Hundreds of proteins accumulate in a tiny volume (Han et al., 2022), creating an extremely high local density. Disrupting phase separation with aliphatic alcohols shrinks the nanoclusters formed by scaffold proteins like RIM1/2 and Munc13, and dysregulates action potential-dependent neurotransmission while largely sparing spontaneous neurotransmission (Guzikowski and Kavalali, 2024). Therefore, nanoclusters are not just small condensates formed by phase separation; they are dynamic nanoclusters with precise binding mechanisms and a functionally specific manner to regulate synaptic transmission.

## FURTHER DIRECTION AND PERSPECTIVE

Synaptopathy refers to the disruption of synaptic structure and function that is central to neurodegenerative and psychiatric disorders and may represent a key determinant of these brain disorders (Brose et al., 2010). Over the past few years, our understanding of how Nlgns and their binding partners regulate synaptic function at the nanoscale has advanced significantly. Yet key challenges remain: uncovering the precise biochemical mechanisms by which those nanoclusters contribute to synapse formation and maturation at the subsynaptic scale and determining how disease-associated genetic mutations alter nanocluster properties. Several outstanding questions about Nlgn-mediated nanoclusters warrant further investigation: (1) How many synaptic proteins are contained within a single synaptic nanocluster? (2) What determines the content, size, and boundary of a nanocluster? (3) How are these synaptic nanoclusters dynamically regulated during development and in response to activity-dependent conditions? Astrocytic Nlgn3 has a puncta-like pattern and is primarily located in the soma region in Bergmann glia in vivo (Qin et al., 2025). Cerebellar synapses had normal densities and functions after the specific deletion of Nlgn3 in astrocytes (Qin et al., 2025). Therefore, astrocytic Nlgn3 does not contribute to the trans-synaptic nanocolumn formation, at least in those brain regions examined. In invertebrates, the localization of CAMs is precisely regulated (Moretto et al., 2019). Given that tagged Nlgn1 mouse lines have been successfully generated to map the endogenous distribution of Nlgn1 (Ducrot et al., 2025, Nozawa et al., 2022, Nozawa et al., 2018), we could apply a similar tagging strategy to other nanocluster proteins. Recent studies have also shown that synaptic adhesion molecules latrophilin-2 and latrophilin-3 bind to distinct ligands, teneurins and FLRT, at different sites of CA1 pyramidal neuron synapses (Sando et al., 2019). Taking advantage of the endogenous tagging system (Matsuda and Oinuma, 2019, Nishiyama et al., 2017), superresolution microscopy (Schermelleh et al., 2019), and conditional knockout models, we are well-positioned to address these critical questions. Answering them will help clarify why functional mammalian brain circuits rely on synaptic CAMs like Nlgns, which orchestrate synaptic strength under both physiological and pathological conditions throughout life.

## Funding and Support

This work was supported by the following funding: Shenzhen Medical Research Fund (Grant No. B2402008 to B.Z.); Major Program of Shenzhen Bay Laboratory (S241101002 to B.Z.); National Natural Science Foundation of China (Grant No. 82022018, 32070958, and 82161138025 to B.Z.); Guangdong Pearl River Funding to B.Z.; Shenzhen-Hong Kong Institute of Brain Science-Shenzhen Fundamental Research Institutions (2024SHIBS0004).

## Author contributions

All authors listed have made a substantial direct and intellectual contribution to the work and have approved it for publication.

## Declaration of Competing Interests

The authors declare that they have no known competing financial interests or personal relationships that could have appeared to influence the work reported in this paper.
